# Clinical Epidemiology of 7126 Melioidosis Patients in Thailand and the Implications for a National Notifiable Diseases Surveillance System

**DOI:** 10.1093/ofid/ofz498

**Published:** 2019-11-19

**Authors:** Viriya Hantrakun, Somkid Kongyu, Preeyarach Klaytong, Sittikorn Rongsumlee, Nicholas P J Day, Sharon J Peacock, Soawapak Hinjoy, Direk Limmathurotsakul

**Affiliations:** 1 Mahidol-Oxford Tropical Medicine Research Unit (MORU), Faculty of Tropical Medicine, Mahidol University, Bangkok, Thailand; 2 Epidemiology Division, Department of Disease Control, Ministry of Public Health, Nonthaburi, Thailand; 3 Centre for Tropical Medicine and Global Health, Nuffield Department of Clinical Medicine, Old Road Campus, University of Oxford, Oxford, United Kingdom; 4 Department of Medicine, University of Cambridge, Cambridge, United Kingdom; 5 Office of International Cooperation, Department of Disease Control, Ministry of Public Health, Nonthaburi, Thailand; 6 Department of Tropical Hygiene, Faculty of Tropical Medicine, Mahidol University, Bangkok, Thailand

**Keywords:** notifiable diseases, surveillance system, melioidosis, *Burkholderia pseudomallei*, epidemiology

## Abstract

**Background:**

National notifiable diseases surveillance system (NNDSS) data in developing countries are usually incomplete, yet the total number of fatal cases reported is commonly used in national priority-setting. Melioidosis, an infectious disease caused by *Burkholderia pseudomallei*, is largely underrecognized by policy-makers due to the underreporting of fatal cases via the NNDSS.

**Methods:**

Collaborating with the Epidemiology Division (ED), Ministry of Public Health (MoPH), we conducted a retrospective study to determine the incidence and mortality of melioidosis cases already identified by clinical microbiology laboratories nationwide. A case of melioidosis was defined as a patient with any clinical specimen culture positive for *B. pseudomallei.* Routinely available microbiology and hospital databases of secondary care and tertiary care hospitals, the national death registry, and NNDSS data were obtained for analysis.

**Results:**

A total of 7126 culture-confirmed melioidosis patients were identified from 2012 to 2015 in 60 hospitals countrywide. The total number of cases diagnosed in Northeast, Central, South, East, North, and West Thailand were 5475, 536, 374, 364, 358, and 19 cases, respectively. The overall 30-day mortality was 39% (2805/7126). Only 126 (4%) deaths were reported to the NNDSS. Age, presentation with bacteremia and pneumonia, prevalence of diabetes, and 30-day mortality differed by geographical region (all *P* < .001). The ED at MoPH has agreed to include the findings of our study in the next annual report of the NNDSS.

**Conclusions:**

Melioidosis is an important cause of death in Thailand nationwide, and its clinical epidemiology may be different by region. In developing countries, NNDSS data can be supplemented by integrating information from readily available routine data sets.

## Teaser

Using readily available microbiology and hospital datasets from 60 secondary-care or tertiary-care hospitals from 2012 to 2015 in Thailand, we identified 2,805 fatal culture-confirmed melioidosis cases. In developing countries, integrated information from routine databases could supplement the National Notifiable Diseases Surveillance System data.

National notifiable diseases surveillance system (NNDSS) data are a key part of public health decision-making in all countries, including priority-setting, planning, resource mobilization and allocation, and monitoring and evaluation of disease prevention and control programs [1]. However, incomplete NNDSS data frequently affect priority-setting and actions by policy-makers, particularly with regards to bacterial diseases in low- and middle-income countries (LMICs) [2–4]. In high-income countries, the completeness of NNDSS data can range from 6% to 99%, and invasive bacterial infections are less likely to be reported compared with AIDS and tuberculosis [5, 6]. One of the solutions for high-income countries is the use of an automated computerized system to capture a combination of case data and laboratory data, create reports, and submit the reports to the responsible authority [7–10]. Affordable solutions to improve the completeness and accuracy of NNDSS data in LMICs are still needed [11, 12].

Melioidosis, an often fatal infectious disease caused by the Gram-negative bacterium *Burkholderia pseudomallei,* is endemic in tropical developing countries [13, 14]. Humans usually acquire melioidosis from *B. pseudomallei* in the environment via skin inoculation, ingestion, and inhalation. Diabetes is the most common clinical risk factor. The majority of patients present with sepsis with or without pneumonia or localized abscesses [15]. The mortality of melioidosis cases ranges from 10% to 63% [14, 16–18]. A modeling study estimated that there are about 165 000 melioidosis cases per year worldwide, of which 89 000 (54%) die [13]. Melioidosis is difficult to diagnose due to nonspecific clinical manifestations and a relative lack of microbiological laboratories in tropical developing countries [14]. The gold standard for the diagnosis of melioidosis is culture [19]. *B. pseudomallei* is not part of the normal human flora, and its isolation from any clinical sample is regarded as diagnostic of melioidosis. An indirect hemagglutination assay (IHA), which detects crude antibodies raised against *B. pseudomallei*, is neither sensitive nor specific, and it is not recommended for the diagnosis of melioidosis in disease-endemic regions [19].

Although the capacity and utilization of microbiological laboratories in public referral hospitals in Thailand are high [20], the national burden and epidemiology of melioidosis remain poorly understood. The NNDSS was established in Thailand in 1968, and melioidosis has been a notifiable disease since 2002 [2]. About 10 melioidosis deaths have been formally reported to the NNDSS each year [2]. However, a single hospital in Northeast Thailand continuously publishes scientific papers reporting about 100 fatal melioidosis cases each year [17, 21]. A modeling study estimated that there could be about 2800 fatal melioidosis cases annually in the country [13]. The low numbers of deaths from melioidosis reported to the NNDSS has meant that melioidosis has not been prioritized by the Ministry of Public Health (MoPH) in Thailand [2]. Here, we aim to determine the incidence, mortality, and clinical epidemiology of melioidosis cases already diagnosed by routine clinical microbiological laboratories in all secondary care and tertiary care hospitals in Thailand from 2012 to 2015, compare our findings with NNDSS data, and supplement the annual report of the NNDSS with our findings.

## METHODS

### Study Area and Population

In 2012, Thailand had a population of 64.4 million, consisted of 77 provinces, and covered 513 120 km^2^. The country can be divided into 6 geographical regions, comprising Northeast, North, East, West, South, and Central [22]. Thai health care services are delivered by multiple levels of health care facilities [23]. In each province, there are primary care units (PCUs) located in subdistricts, community hospitals (district level), and at least 1 general or regional hospital. Severely ill patients presenting to PCUs and community hospitals are often referred to general hospitals (acting as secondary care hospitals) or regional hospitals (acting as tertiary care hospitals). In 2012, there were 68 public general hospitals and 28 public regional hospitals in Thailand [24]. Unlike PCUs and community hospitals, these are equipped with a microbiology laboratory capable of performing bacterial culture using standard methodologies for bacterial identification and susceptibility testing provided by the Bureau of Laboratory Quality and Standards, MoPH, Thailand [25].

### Study Design and Source of Data

Collaborating with the Epidemiology Division (ED) of the Department of Disease Control, MoPH, Thailand, we conducted a retrospective, multicenter surveillance study in all public general and regional hospitals in Thailand. From the hospitals that agreed to participate, data were collected from microbiology and hospital databases between January 2012 and December 2015. Hospital number (HN) and admission number (AN) were used as a record linkage between the 2 databases and to identify individuals who had repeat admissions. Diagnoses in the hospital data were recorded using 10th revision of the International Classification of Disease (ICD) codes. Date of death and ICD-10 codes was also extracted from these data.

It is a common in Thailand for terminally ill patients to be discharged from hospital to be allowed to die at home [26, 27], so 30-day mortality was verified using the death registry data of the Ministry of Public Health, Thailand. NNDSS data were obtained from the ED, MoPH. The data variables included province, type of health care facilities, total number of cases, and total number of deaths.

### Definitions

Culture-confirmed melioidosis was defined as a patient with a culture positive for *B. pseudomallei* from any clinical specimen. Comorbidities (diabetes mellitus, hypertension, chronic renal failure, chronic obstructive pulmonary disease [COPD], chronic liver disease, HIV, tuberculosis, thalassemia, and malignancy) were defined using ICD-10, Thai edition, codes (Supplementary Table 1) [28]. Bacteremia and bacteriuria were defined as blood and urine cultures positive for *B. pseudomallei*, respectively. Pneumonia was defined using ICD-10 codes or sputum culture positive for the organism. Hepatosplenic abscess, septic arthritis, and osteomyelitis were defined using ICD-10 codes.

Thirty-day mortality was determined on the basis of a record of death within 30 days of admission in the routine hospital database or by a record of death within that period in the national death registry. In-hospital mortality was determined using the discharge status recorded in the hospital admission data for that admission. In the event that a patient had more than 1 episode of admission due to culture-confirmed melioidosis, only the first episode was included in the study.

### Statistical Analysis

The outcomes of interest were incidence and 30-day mortality, and their associations with regions, comorbidities, and clinical manifestations. The incidence rate (per 100 000 population per year) was calculated by dividing the cumulative incidence by the total population in the study province. The reporting completeness was calculated by dividing the total number of fatal cases reported by the total number of fatal cases observed. Interquartile ranges (IQRs) are presented in terms of 25th and 75th percentiles. Binary and continuous variables were compared using the chi-square test and Kruskal-Wallis test, respectively. The risk factors associated with 30-day mortality were evaluated using a univariable and multivariable logistic regression model stratified by hospital. The final multivariable logistic regression models were developed using a purposeful selection method [29]. Poisson regression models were used to assess changes in incidence rates over time and to compare incidence rates among regions. All models were stratified by hospital. A sensitivity analysis was done by evaluating factors associated with in-hospital mortality. All statistical analyses were performed using Stata, version 15.0 (StataCorp LP, College Station, TX, USA).

### Ethical Considerations

Ethical permission for this study was obtained from the Institute for the Development of Human Research Protection, Ministry of Public Health (IHRP 2334/2556), the Ethics Committee of the Faculty of Tropical Medicine, Mahidol University (MUTM 2014-017-01), and the Oxford Tropical Research Ethics Committee, University of Oxford (OXTREC 521-13). Written approval was given by the directors of the hospitals to use their routine hospital database for research. Individual consent was not sought from the patients as this was a retrospective study, and the Ethical and Scientific Review Committees approved the process.

## RESULTS

**Figure 1.**
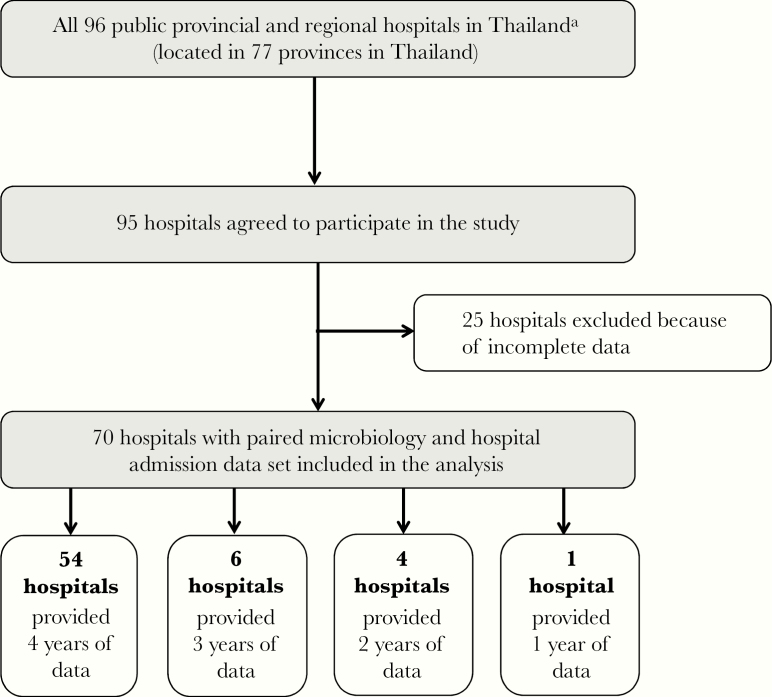
Flowchart of the study. ^a^In 2012, there were 68 public general hospitals (acting as secondary care hospitals) and 28 public regional hospitals (acting as tertiary care hospitals) in Thailand [24].

**Figure 2.**
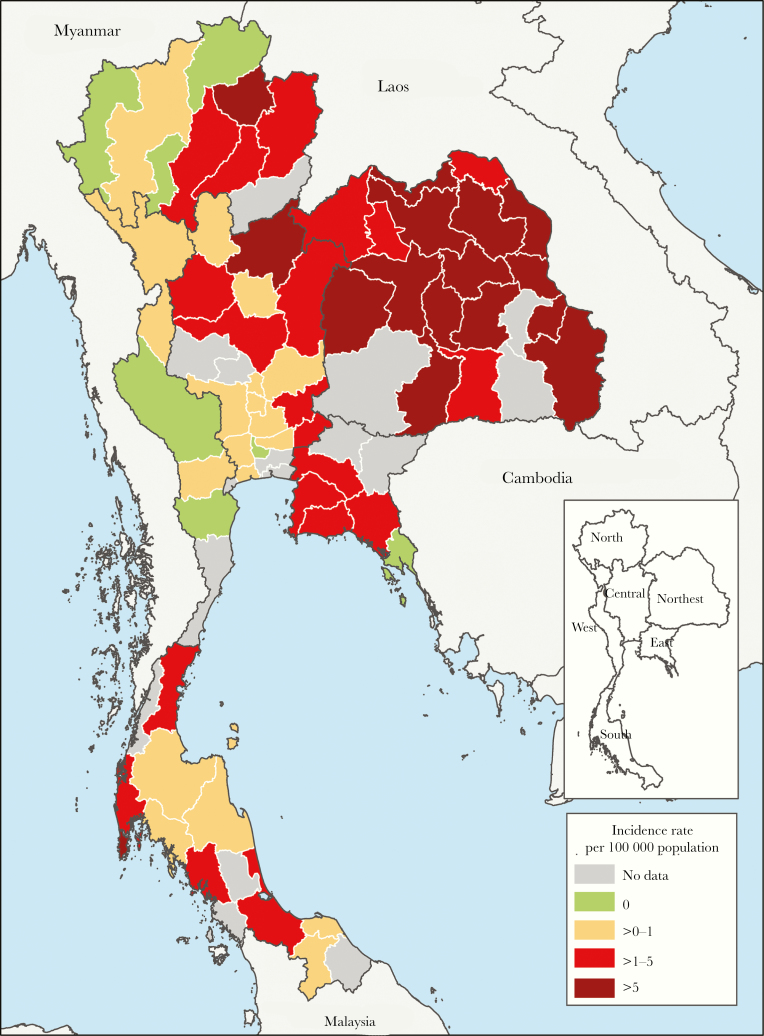
Incidence rates of culture-confirmed melioidosis in Thailand from 2012 to 2015. Provinces are categorized based on incidence rates of culture-confirmed melioidosis observed (dark red, >5 cases per 100 000 population per year; red, >1–5 cases per 100 000 population per year; yellow, >0–1 cases per 100 000 population per year; green, no cases observed; and gray, no data)

Of 96 public general and regional hospitals in Thailand, 95 (99%) agreed to participate in the study (Figure 1). Twenty-five hospitals (26%) were not included in the analysis because either the microbiology or hospital database was not obtained. Seventy hospitals included in the analysis were located in 61 provinces (Figure 2). A total of 54 hospitals (77%) provided data for 4 years (from 2012 to 2015), 6 hospitals (9%) for 3 years, 4 hospitals (6%) for 2 years, and 6 hospitals (8%) for 1 year (see supplementary Table 2).

A total of 8 476 596 admission records from 6 228 644 patients were evaluated, and 7626 admission records had at least 1 clinical sample culture positive for *B. pseudomallei.* Multiple admissions during which *B. pseudomallei* was grown from clinical specimens were noted in 421 patients. Only the first episode of culture-confirmed melioidosis in 7126 patients was included in further analysis.

### Incidence of Melioidosis

The total numbers of culture-confirmed melioidosis cases identified in 2012, 2013, 2014, and 2015 were 1735, 1757, 1932, and 1702, respectively (Table 1). Overall, melioidosis cases were already diagnosed in 60 hospitals located in 52 provinces (Figure 2). The average incidence rate of melioidosis during the 4-year study period was 3.95 per 100 000 population per year and was significantly different by region (*P* < .001). There was no clear trend over the study period. The total number of cases diagnosed in Northeast, Central, South, East, North, and West Thailand were 5475, 536, 374, 364, 358, and 19 cases, respectively. The incidence rate was highest in Northeast Thailand (8.73 per 100 000 population per year) and lowest in West Thailand (0.23 per 100 000 population per year; *P *< .001) (Supplementary Table 3).

**Table 1.** Total Number of Culture-Confirmed Melioidosis Cases Diagnosed by Routine Clinical Microbiology Laboratories in Public Secondary Care and Tertiary Care Hospitals in Thailand in 2012 to 2015

			No. of Culture-Confirmed Melioidosis Cases^b^				
Regions	No. of Participating Hospitals	No. of Provinces^a^	2012	2013	2014	2015	Total
Northeast	17	17	1332	1359	1481	1303	**5475**
Central	21	17	112	142	155	127	**536**
South	12	10	97	75	95	107	**374**
East	5	5	85	84	113	82	**364**
North	9	8	99	93	85	81	**358**
West	6	4	10	4	3	2	**19**
Total	**70**	**61**	1735	1757	1932	1702	**7126**

^a^Eight provinces had the data obtained from more than 1 hospital, including Lopburi (2), Phang Nga (2), Phayao (2), Ratchaburi (3), Saraburi (2), Singburi (2), Songkhla (2), and Suphanburi (2).

^b^Of 70 provincial or regional hospitals included in the study, 65, 58, 64, and 62 provided data for years 2012, 2013, 2014, and 2015, respectively.

### Clinical Epidemiology of Melioidosis

Of the 7126 patients, 4839 (68%) were male, and the median age (IQR; range) was 54 (44.5–63; <1–100) years (Supplementary Table 4). Using ICD-10 codes, we found that the most common comorbidities reported were diabetes mellitus (43%), followed by hypertension (15%) and chronic kidney disease (11%). The most common clinical specimens that were culture positive for *B. pseudomallei* were blood (n = 4910, 69%), sputum (n = 1555, 22%), urine (n = 341, 5%), pleural fluid (n = 92, 1%), cerebrospinal fluid (n = 13, 0.2%), and unidentified pus or fluid (n = 1143, 16%). Using the combination of ICD-10 codes and the microbiology laboratory database, we found that the most common clinical presentation was bacteremia (69%), followed by pneumonia (38%), hepatosplenic abscesses (8%), and bacteriuria (5%).

Age, comorbidities, and clinical presentations of melioidosis in Thailand differed by geographical region (Supplementary Table 4). The median age of patients was highest in North Thailand (57 years) and lowest in West Thailand (48 years; *P* < .001). The prevalence of diabetes mellitus was highest in South Thailand (48%) and lowest in North Thailand (21%; *P* < .001). Presentation with bacteremia was highest in East Thailand (78%) and lowest in West Thailand (63%). Presentation with pneumonia was also highest in East Thailand (46%) and lowest in West Thailand (16%).

### Mortality Involving Melioidosis

A total of 2805 cases died within 30 days of hospital admission, giving a 30-day mortality of 39% (2805/7126). Death in melioidosis patients often occurred rapidly, with 1076 deaths (39%) occurring within the first 2 days of admission, 894 (32%) from day 3 to day 7, and the remaining 835 (30%) after 7 days of admission.

In the univariable logistic regression models, 30-day mortality was associated with older age, comorbidities, clinical presentation, and region (Supplementary Table 5). In the final multivariable model, 30-day mortality was associated with the comorbidities of chronic kidney disease and liver disease, and presentation with bacteremia, pneumonia, and bacteriuria (Table 2). Male gender, comorbidities of diabetes and thalassemia, and presentation with hepatosplenic abscesses, septic arthritis, and osteomyelitis were associated with survival. Sensitivity analysis showed that factors associated with in-hospital mortality were similar to factors associated with 30-day mortality, except that some *P* values were slightly higher (Supplementary Tables 6 and 7).

**Table 2.** Factors Associated With 30-Day Mortality in 7126 Culture-Confirmed Melioidosis Cases in 2012–2015, by Multivariable Logistic Regression Model, Stratified by Hospital

Baseline Characteristics	Died (n = 2805)	Survived (n = 4321)	Adjusted Odds Ratio(95% CI)	*P*
Gender (male), No. (%)	1908 (68.0)	2931 (67.8)	0.84 (0.74–0.94)	.004
Age, median (IQR), y	56 (46–65)	53 (43–61)	1.01 (1.01–1.02)	<.001
Comorbidities,^a^ No. (%)				
Liver disease	371 (13.2)	290 (6.7)	1.89 (1.57–2.28)	<.001
Chronic kidney disease	410 (14.6)	405 (9.4)	1.54 (1.30–1.83)	<.001
Thalassemia	36 (1.3)	115 (2.7)	0.60 (0.38–0.92)	.02
Diabetes mellitus	1061 (37.8)	1984 (45.9)	0.57 (0.50–0.64)	<.001
Clinical manifestations, No. (%)				
Bacteremia^b^	2391 (85.2)	2519 (58.3)	5.66 (4.93–6.51)	<.001
Pneumonia^c^	1574 (56.1)	1131 (26.2)	4.44 (3.94–4.99)	<.001
Bacteriuria^d^	209 (7.5)	132 (3.1)	3.14 (2.41–4.09)	<.001
Hepatosplenic abscess^e^	100 (3.6)	480 (11.1)	0.35 (0.28–0.45)	<.001
Septic arthritis^e^	85 (3.0)	300 (6.9)	0.61 (0.46–0.81)	.001
Ostemomyelitis^e^	6 (0.2)	57 (1.3)	0.36 (0.14–0.91)	.03
Regions, No. (%)				
Northeast	2190 (78.1)	3285 (76.0)	1	.05
Central	215 (7.7)	321 (7.4)	0.95 (0.72–1.25)	
East	158 (5.6)	206 (4.8)	0.91 (0.66–1.25)	
North	111 (4.0)	247 (5.7)	0.60 (0.43–0.84)	
South	129 (4.6)	245 (5.7)	0.87 (0.64–1.17)	
West	2 (0.1)	17 (0.4)	0.26 (0.06–1.24)	

Abbreviations: CI, confidence interval; IQR, interquartile range.

^a^Comorbidities identified by using the ICD-10 codes listed in Supplementary Table 1.

^b^Blood culture positive for *B. pseudomallei*.

^c^Using ICD-10 codes or sputum culture positive for *B. pseudomallei*.

^d^Urine culture positive for *B. pseudomallei*.

^e^Using ICD-10 codes.

### Comparison Between Hospital Data and NNDSS Data

The total number of melioidosis cases reported to the NNDSS during the study period was 12 305, of which 141 were reported as fatal cases (Table 3). Of 2805 fatal melioidosis cases identified by the microbiology and hospital databases of the participating hospitals during the study period, 126 were reported as fatal cases to Report 506, giving a completeness of 4% (126/2805).

**Table 3.** Comparison Between Incidences and Mortalities of Melioidosis Diagnosed by Microbiology Laboratories and Those Officially Reported to the National Notifiable Disease Surveillance System (Report 506) in Thailand From 2012 to 2015

		Culture-Confirmed Melioidosis^a^		Report 506 Data^b^	
Year	Type of Hospital	No. of Cases Diagnosed	No. of Mortality Outcome	No. of Cases Reported	No. of Mortality Outcome Reported
2012	PCUs or community hospitals	NA	NA	2426	2
	Regional or general hospitals not included in the study	NA	NA	259	7
	Regional or general hospital included in the study	1735	683	1018	4
2013	PCUs or community hospitals	NA	NA	1821	0
	Regional or general hospitals not included in the study	NA	NA	210	1
	Regional or general hospital included in the study	1757	737	799	3
2014	PCUs or community hospitals	NA	NA	1677	3
	Regional or general hospitals not included in the study	NA	NA	174	1
	Regional or general hospital included in the study	1932	750	695	8
2015	PCUs or community hospitals	NA	NA	2042	1
	Regional or general hospitals not included in the study	NA	NA	217	0
	Regional or general hospital included in the study	1702	635	967	111 ^c^

Abbreviations: IHA, indirect hemagglutination assay; PCU, primary care unit.

^a^Seventy of 96 public general and regional hospitals in Thailand participated in the study.

^b^Based on the national notifiable disease surveillance system in Thailand. Both probable and confirmed melioidosis cases are reported. Probable cases are defined as clinically compatible illness with IHA titer ≥1:160 or IFA >1:400. Confirmed melioidosis cases are defined as clinically compatible illness with any clinical specimen culture positive for *B. pseudomallei* or a 4-fold rise in IHA or IFA.

^c^In 2015, 107 of 111 fatal cases (96%) were reported by a single regional hospital, Sunpasitthiprasong Hospital, Ubon Ratchathani, in Northeast Thailand.

### Policy-Maker Engagement

The findings of our study were reported to the ED, MoPH, which agreed to include the findings in the next annual report of the NNDSS.

## Discussion

Using routine microbiology and hospital databases, we show that in Thailand each year, about 1700 culture-confirmed melioidosis cases are diagnosed, of whom approximately 700 die. Only about 4% of the deaths were reported to the NNDSS Thailand. Integrating information from readily available microbiology and hospital data can reveal the burden of underreported notifiable diseases. This information could support priority-setting by policy-makers in LMICs. We propose, therefore, that integrating information from readily available data sets to improve national statistics and NNDSS data in LMICs should be considered and implemented.

We expect that including the findings of our study in the next annual report of the NNDSS could support initiatives toward a National Programme for Melioidosis to improve awareness, surveillance, diagnosis, treatment, and prevention of melioidosis by MoPH Thailand. Our finding of deaths involving melioidosis (about 700 fatal cases per year) is much higher than the mortalities involving dengue infection (about 100 fatal cases per year) shown in the annual report of the NNDSS Thailand [2]. In Thailand, dengue infection is considered high priority by policy-makers. There is a National Programme for Dengue Prevention Control focused on empowering individuals and communities for source reduction, health promotion, medical services, multisectoral networking, and enhancing capacity-building [30]. These activities improve dengue diagnosis and reporting, and most, if not all, diagnosed fatal cases are reported via the NNDSS.

Underreporting to the NNDSS could occur for a range of reasons. First, persons who are responsible for reporting notifiable diseases in many hospitals with microbiology laboratories (including epidemiologists, nurses, and doctors [31]) do not know that they should confirm a fatal outcome of all culture-confirmed melioidosis cases and report the death of any patients with culture-confirmed melioidosis to the NNDSS [2]. Second, laboratory isolation and identification of *B. pseudomallei* can take from 2 to 7 days, and many melioidosis cases could die before the culture results. In such cases, nurses and physicians would not be aware of the causative pathogen, and epidemiologists in the hospitals would not be informed and would not report the cases to the NNDSS [31]. Third, the definition of melioidosis used for the NNDSS Thailand is broad. The NNDSS Thailand recommends that both probable and confirmed melioidosis cases be reported [2]. Probable cases are defined as clinically compatible illness with an IHA titer ≥1:160 or immunofluorescence antibody test (IFA) >1:400. Confirmed melioidosis cases are defined as clinically compatible illness with any clinical specimen culture positive for *B. pseudomallei* or a 4-fold rise in IHA or IFA. However, IFA and IHA are not recommended for diagnosing melioidosis in disease-endemic areas; these tests are neither sensitive nor specific [19]. More than 60% of melioidosis cases reported to Report 506 are from PCUs or community hospitals that do not have microbiology laboratories, and a proportion of these reported cases are likely to be false-positive cases (ie, cases who do not have melioidosis but tested IFA or IHA positive due to previous exposure to environmental *B. pseudomallei* [19]).

Evaluating the incidence, mortality, and clinical epidemiology of a notifiable disease among provinces could give new information about diseases and highlight areas where diagnosis or reporting systems may need additional investigation. For example, culture-confirmed melioidosis cases were not identified in 9 provinces (10 hospitals) participating in our study, but a high incidence of culture-confirmed melioidosis cases was observed in neighboring provinces in our study. It is possible that *B. pseudomallei* may be misidentified as *Pseudomonas* spp. or that there may be contaminants in the laboratories in those provinces. This suggests that evaluation of protocols and operating procedures in microbiological laboratories in these provinces may be warranted.

The differences in clinical presentations and mortality of melioidosis across geographical regions could be due to several reasons, including differing characteristics of the baseline population, differences in the distribution of environmental *B. pseudomallei* [32, 33], virulence characteristics of *B. pseudomallei* [34], variation in risk of exposure and route of infection related to occupational activities [14, 35], and disparity among practices of physicians and clinical microbiological laboratories in the region. Selective culture media for *B. pseudomallei*, which can increase the sensitivity of bacterial isolation from nonsterile specimens such as sputum and urine, is only used in a limited number of hospitals in Northeast Thailand [2]. Variation in clinical presentations and comorbidities could also be due to different practices in recording ICD-10 codes by trained ICD coders or attending physicians in each region. This suggests that training for laboratory personnel and clinicians, informing clinicians of possible variation in clinical presentation of melioidosis cases, and workshops to improve communication between laboratory personnel, clinicians, ICD-10 coders, and persons responsible for NNDSS reporting should be provided countrywide. Further studies on differences in clinical presentations and mortality of melioidosis across regions are also required.

The lower overall mortality of patients with diabetes could be due to the use of glibenclamide [14, 36]. This has an anti-inflammatory effect, and patients taking glibenclamide before hospital admission have attenuated inflammatory responses [14, 36]. The lower overall mortality of patients with thalassemia could be due to early diagnosis of melioidosis in patients with a major underlying disease, unknown reasons (eg, increasing utilization of iron chelation therapy in Thailand), or residual confounding factors. Patients with thalassemia were reported to have a high mortality (59%, 16/28) in Sabah, Malaysia, and the incidence of melioidosis has decreased considerably since the universal availability of iron chelation therapy [37].

We propose a set of low-cost actions to improve NNDSS data in LMICs, including (1) routinely utilizing all laboratory databases (including microbiology, serology, and rapid diagnostic test databases) and hospital databases from all public and private hospitals to supplement NNDSS data of all notifiable diseases, (2) providing training to laboratory personnel countrywide to improve the sensitivity and accuracy of laboratory-diagnosed cases, (3) raising awareness among health care providers about diagnostic criteria and requirements for reporting the final outcome of all cases with notifiable diseases, and (4) updating criteria for diagnosing and reporting notifiable diseases. For example, patients culture positive for *B. pseuodmallei* should be defined as “culture-confirmed melioidosis cases” and reported to the NNDSS, similar to systems in Singapore, Australia, and Taiwan [38–40]. Melioidosis cases diagnosed based on the IHA or IFA without culture confirmation should be defined as “possible melioidosis cases” when reported to the NNDSS Thailand. These actions are being implemented or discussed with the ED, MoPH Thailand, in conjunction with the current NNDSS.

The difference between the observed 701 fatal culture-confirmed melioidosis cases pear year in this study and the predicted 2838 fatal melioidosis cases per year in the previous modeling study [13] could be due to multiple reasons. First, only 74% of public general and regional hospitals in Thailand were included in the study, and only 77% of those hospitals provided data for all 4 years. Therefore, our data represented only about two-thirds of the already diagnosed melioidosis patients in Thailand. Second, it is possible that the participating hospitals may still misidentify a proportion of the *B. pseudomallei* isolates as contaminants or other bacteria [19]. Although it is possible that other bacteria could be misidentified as *B. pseudomallei*, we believe that it is rare based on the increasing clinical information and bacterial confirmation of melioidosis cases in all regions [2]. Third, the modeling study was based on data from large hospitals with research facilities, where the blood culture utilization rate is already high [20] and selective culture media for *B. pseudomallei* are used for nonsterile specimens collected from melioidosis-suspected cases [13, 19]. It is likely that some public general and regional hospitals will find more melioidosis cases if culturing practices change and selective culture media are used for melioidosis-suspected cases [16, 17]. Therefore, the number of cases and deaths from melioidosis reported here could represent a minimum estimate. Fourth, the previous model was imprecise, as shown by the wide 95% credible interval of the predicted mortality (1259 to 6678) due to limited data availability at that time [13]. The model could be revised and improved by using increasingly available data in the future.

The limitations of this study are that private hospitals, specialized hospitals such as military hospitals and psychiatric hospitals, hospitals in Bangkok, and university hospitals were not included in the study. The lack of culture-confirmed melioidosis identified in certain provinces should be interpreted with caution; an absence of risk for melioidosis acquisition in these areas should not be implied.

In conclusion, the high number of deaths from melioidosis reported in our study provides policy-makers with the evidence they need to accord a high priority to melioidosis as a major health problem in Thailand. Integrating information from readily available microbiology and hospital databases could be used to generate such information, supplement NNDSS data, and support priority-setting for policy-makers in LMICs.

## Supplementary Data

Supplementary materials are available at *Open Forum Infectious Diseases* online. Consisting of data provided by the authors to benefit the reader, the posted materials are not copyedited and are the sole responsibility of the authors, so questions or comments should be addressed to the corresponding author.

ofz498_suppl_Supplementary_Tables
