# Supplementary material for: Clinical Epidemiology of 7126 Melioidosis Patients in Thailand and the Implications for a National Notifiable Diseases Surveillance System
Source: Open Forum Infect Dis. 2019 Nov 19;6(12):ofz498. doi: 10.1093/ofid/ofz498 (PMC7020769; doi:10.1093/ofid/ofz498)
Supplement: ofz498_suppl_Supplementary_Tables [file ofz498_suppl_supplementary_tables.docx]

# Clinical Epidemiology of 7,126 Melioidosis Patients in Thailand and the implications for a National Notifiable Diseases Surveillance System

Viriya Hantrakun, PhD^1^; Somkid Kongyu, MSc^2^; Preeyarach Klaytong, MSc^1^; Sittikorn Rongsumlee, MSc^1^; Nicholas PJ Day, FRCP^1,3^; Sharon J Peacock, FRCP^4^; Soawapak Hinjoy, DrPH^2,5^; Direk Limmathurotsakul, PhD^1,3,6^

^1^ Mahidol-Oxford Tropical Medicines Research Unit (MORU), Faculty of Tropical Medicine, Mahidol University, Bangkok, Thailand

^2^ Epidemiology Division, Department of Disease Control, Ministry of Public Health, Nonthaburi, Thailand

^3^ Centre for Tropical Medicine and Global Health, Nuffield Department of Clinical Medicine, Old Road Campus, University of Oxford, Oxford, United Kingdom

^4^ Department of Medicine, University of Cambridge, Cambridge, United Kingdom

^5^ Office of International Cooperation Department of Disease Control, Ministry of Public Health, Nonthaburi, Thailand

^6^ Department of Tropical Hygiene, Faculty of Tropical Medicine, Mahidol University, Bangkok, Thailand

Supplementary Text

Supplementary Results

Mortality involving melioidosis

A total of 2,805 cases died within 30 days of hospital admission, giving a 30-day mortality of 39% (2,805/7,126). Death in melioidosis patients occurred rapidly, with 1,076 deaths (39%) occurring within the first two days of admission, 894 (32%) from day 3 to day 7, and the remaining 835 (30%) after 7 days of admission. Fifty nine percent of deaths (1,663/2,805) occurred in hospital, while the remainder (41%; 1,142/2,805) occurred after hospital discharge with a hospital record of refusal of treatment (75%; 863/1,142), an improvement in condition (19%; 212/1,142), transfer to other hospitals (5%; 58/1,142) or no record of outcome at time of discharge (0.8%; 9/1,142). Among 1,142 patients who died after hospital discharge, 890 (78%) died within the first two days of hospital discharge, 123 (11%) from day 3 to day 7, and the remaining 129 (11%) after 7 days of hospital discharge.

The mortality rate of culture-confirmed melioidosis in Thailand was 1.6 per 100,000 population per year, and was significantly different by regions (p<0.001). The total number of fatal cases in Northeast, Central, South, East, North, and West Thailand were 2,190, 215, 129, 158, 111 and 2 cases, respectively. The mortality rate was highest in Northeast (3.5 per 100,000 population per year) and lowest in West Thailand (0.02 per 100,000 population per year).

Sensitivity Analysis

A sensitivity analysis was conducted to evaluate factors associated with in-hospital mortality. using data from the year 2012-2015. A total of 1,742 cases had a discharge status of death for that admission; including 1663 cases (95%) who died in hospital within 30 days of admission as described above and another 79 cases (5%) who died in hospital after 30 days of admission. This gave a in-hospital mortality of 24% (1,742/7,126). In the univariable model (supplementary Table S6), region was associated with in-hospital mortality (p<0.001). In a multivariable logistic regression model, in-hospital mortality was significantly associated with underlying liver disease, presentations with bacteremia, pneumonia and bacteriuria, and region (supplementary Table S7). The underlying diseases of diabetes and thalassemia, and presentations with hepatosplenic abscess, septic arthritis and osteomyelitis were associated with survival discharge.

Supplementary tables

Supplementary Table S1 List of ICD-10 (Thai Modification 2012) codes used to determine underlying diseases and clinical manifestations

| **Factors** | **ICD-10 codes** |
| --- | --- |
| **Comorbidities:** |  |
| • Diabetes mellitus | E10-14 |
| • Hypertension | I10, I15.8-I15.9 |
| • Chronic kidney disease | N18.1-N18.5, 189 |
| • COPD | J42, J43, J44.0-J44.1, J44.8-J44.9, J47 |
| • Liver disease | K70, K72-K74, K75.1-K76, K77, B18 |
| • HIV | B20-B23, B24 |
| • Pulmonary tuberculosis | A15.0-A15.9, A16.0-A16.9, O98.0, P37.0, B90.9 |
| • Thalassemia | D56.0-D56.4, D56.8-D56.9 |
| • Malignancy | C00-C75, C81-C96, C76-C80, C97 |
| **Clinical manifestations:** |  |
| • Pneumonia | B01.2, B05.2, B20.6, J10.0, J11.0, J12.0-J12.2, J12.8-J12.9, J13-J14, J15.0-J15.7, J15.81, J15.88, J16.8, J17.0-J17.3, J17.8, J18.0-J18.2, J18.8-J18.9, J22, J44.0, J85.1-J85.2, P23.0-P23.6, and P23.8-P23.9 |
| • Hepatosplenic abscesses | A06.4, D73.3 and K75.0 |
| • Septic arthritis | M00.0-M00.2 and M00.8-M01.0 |
| • Osteomyelitis | M46.2, M86 |

Supplementary Table S2 Incidence and in-hospital mortality of culture-confirmed melioidosis cases diagnosed at 70 hospitals in Thailand from 2012 to 2015

| **No** | **Hospital** | **Province** | **Region** | **Year of data included** | | **Culture confirmed melioidosis (primary episode)** | | |
| --- | --- | --- | --- | --- | --- | --- | --- | --- |
|  |  |  |  |  |  | **No of cases** | **No of in- hospital deaths** | **CFR^1^** |
| 1 | Ang Thong hospital | Angthong | Central | 2012-2015 | | 1 | 1 | 100% |
| 2 | Ban Mi hospital | Lopburi | Central | 2014-2015 | | 5 | 3 | 60% |
| 3 | Buddhachinaraj hospital | Phitsanulok | Central | 2012-2015 | | 186 | 61 | 33% |
| 4 | Chao Phaya Yommarat hospital | Suphan Buri | Central | 2012-2015 | | 22 | 10 | 45% |
| 5 | Inburi Hospital | Singburi | Central | 2012-2015 | | 0 | 0 | 0% |
| 6 | Kamphaeng Phet hospital | Kamphaengphet | Central | 2012-2015 | | 66 | 26 | 39% |
| 7 | Kingnarai hospital | Lopburi | Central | 2012 | | 1 | 1 | 100% |
| 8 | Nakhon Nayok hospital | Nakhonnayok | Central | 2012,2014 | | 24 | 9 | 38% |
| 9 | Nakhon Pathom hospital | Nakhonpathom | Central | 2012-2015 | | 12 | 2 | 17% |
| 10 | Pathum Thani hospital | Pathumthani | Central | 2012-2015 | | 1 | 1 | 100% |
| 11 | Phanakorn Si Ayutthaya hospital | Ayutthaya | Central | 2013-2015 | | 3 | 1 | 33% |
| 12 | Phetchabun hospital | Phetchabun | Central | 2012-2015 | | 53 | 19 | 36% |
| 13 | Phichit Hospital | Phichit | Central | 2012-2015 | | 2 | 1 | 50% |
| 14 | Phra Phutthabat Hospital | Saraburi | Central | 2012-2015 | | 3 | 2 | 67% |
| 15 | Pranangklao Hospital | Nonthaburi | Central | 2012-2015 | | 0 | 0 | 0% |
| 16 | Samut Sakhon hospital | Samutsakhon | Central | 2012 | | 1 | 0 | 0% |
| 17 | Saraburi hospital | Saraburi | Central | 2012-2015 | | 42 | 12 | 29% |
| 18 | Sawan Pracha Rak hospital | Nakhonsawan | Central | 2012-2015 | | 100 | 42 | 42% |
| 19 | Singburi hospital | Singburi | Central | 2013-2015 | | 1 | 1 | 100% |
| 20 | Somdejprasangkharach XVII hospital | Suphan Buri | Central | 2012-2015 | | 3 | 0 | 0% |
| 21 | Srisangworn hospital | Sukhothai | Central | 2012-2015 | | 10 | 4 | 40% |
| 22 | Bhuda Sothon hospital | Chachoengsao | East | 2012-2015 | | 113 | 47 | 42% |
| 23 | Chon Buri hospital | Chonburi | East | 2012-2015 | | 107 | 59 | 55% |
| 24 | Prapokkloa hospital | Chanthaburi | East | 2012-2015 | | 68 | 19 | 28% |
| 25 | Rayong hospital | Rayong | East | 2012-2015 | | 76 | 36 | 47% |
| 26 | Trat Hospital | Trat | East | 2012 | | 0 | 0 | 0% |
| 27 | Chiangkhum hospital | Phayao | North | 2012-2015 | | 44 | 12 | 27% |
| 28 | Chiangrai Prachanukroh Hospital | Chiangrai | North | 2012-2015 | | 0 | 0 | 0% |
| 29 | Lampang hospital | Lampang | North | 2012-2015 | | 140 | 24 | 17% |
| 30 | Lamphun Hospital | Lamphun | North | 2012-2015 | | 0 | 0 | 0% |
| 31 | Nakornping hospital | Chiangmai | North | 2012-2015 | | 3 | 0 | 0% |
| 32 | Nan hospital | Nan | North | 2012-2015 | | 90 | 24 | 27% |
| 33 | Phayao hospital | Phayao | North | 2012-2015 | | 62 | 13 | 21% |
| 34 | Phrae Hospital | Phrae | North | 2013-2015 | | 19 | 3 | 16% |
| 35 | Srisangwan Hospital | Maehongson | North | 2014-2015 | | 0 | 0 | 0% |
| 36 | Amnat Charoen hospital | Amnat Charoen | Northeast | 2012 | | 69 | 8 | 12% |
| 37 | Buengkan Hospital | Buengkan | Northeast | 2014-2015 | | 28 | 9 | 32% |
| 38 | Buri Ram hospital | Buriram | Northeast | 2012-2015 | | 372 | 105 | 28% |
| 39 | Chaiyaphum hospital | Chaiyaphum | Northeast | 2012 | | 63 | 24 | 38% |
| 40 | Kalasin hospital | Kalasin | Northeast | 2012-2015 | | 289 | 79 | 27% |
| 41 | Khon Kaen hospital | Khon Kaen | Northeast | 2012-2015 | | 506 | 58 | 11% |
| 42 | Loei hospital | Loei | Northeast | 2012-2015 | | 61 | 18 | 30% |
| 43 | Maha Sarakham hospital | Mahasarakham | Northeast | 2012-2015 | | 258 | 54 | 21% |
| 44 | Mukdahan hospital | Mukdahan | Northeast | 2012-2015 | | 125 | 19 | 15% |
| 45 | Nakhon Phanom hospital | Nakhonphanom | Northeast | 2012-2015 | | 358 | 78 | 22% |
| 46 | Nong Bua Lamphu hospital | Nongbualamphu | Northeast | 2013-2015 | | 35 | 6 | 17% |
| 47 | Nong Khai hospital | Nongkhai | Northeast | 2012-2015 | | 108 | 14 | 13% |
| 48 | Roi Et hospital | Roiet | Northeast | 2012-2015 | | 496 | 104 | 21% |
| 49 | Sakonnakhon hospital | Sakonnakhon | Northeast | 2012-2015 | | 708 | 197 | 28% |
| 50 | Sappasitthiprasong hospital | Ubonratchathani | Northeast | 2012-2015 | | 1219 | 234 | 19% |
| 51 | Surin hospital | Surin | Northeast | 2012-2015 | | 124 | 31 | 25% |
| 52 | Udon Thani hospital | Udonthani | Northeast | 2012-2015 | | 656 | 158 | 24% |
| 53 | Chumphonkhetudomsakdi hospital | Chumphon | South | 2012-2015 | | 33 | 13 | 39% |
| 54 | Hatyai hospital | Songkhla | South | 2012-2015 | | 59 | 20 | 34% |
| 55 | Krabi hospital | Krabi | South | 2012-2015 | | 18 | 3 | 17% |
| 56 | Maharaj Nakhonsithammarat hospital | Nakhonsithammarat | South | 2012-2015 | | 58 | 25 | 43% |
| 57 | Pattani Hospital | Pattani | South | 2012-2015 | | 8 | 1 | 13% |
| 58 | Phang Nga hospital | Phang Nga | South | 2012-2013,2015 | | 3 | 1 | 33% |
| 59 | Songkhla hospital | Songkhla | South | 2012-2015 | | 29 | 11 | 38% |
| 60 | Surat Thani hospital | Suratthani | South | 2012-2015 | | 13 | 2 | 15% |
| 61 | Takuapa hospital | Phang Nga | South | 2014-2015 | | 15 | 5 | 33% |
| 62 | Trang hospital | Trang | South | 2012-2015 | | 39 | 3 | 8% |
| 63 | Vachira Phuket hospital | Phuket | South | 2012-2015 | | 79 | 22 | 28% |
| 64 | Yala hospital | Yala | South | 2012-2015 | | 20 | 4 | 20% |
| 65 | Banpong Hospital | Ratchaburi | West | 2012-2015 | | 0 | 0 | 0% |
| 66 | Damnoensaduak Hospital | Ratchaburi | West | 2012-2015 | | 0 | 0 | 0% |
| 67 | King Taksinmaharaj Memorial hospital | Tak | West | 2012-2015 | | 18 | 3 | 17% |
| 68 | Pahonpol Payuha Sena Hospital | Kanchanaburi | West | 2012 | | 0 | 0 | 0% |
| 69 | Photharam Hospital | Ratchaburi | West | 2012-2015 | | 1 | 0 | 0% |
| 70 | Prajomklao Hospital | Phetchaburi | West | 2012-2015 | | 0 | 0 | 0% |
|  |  |  |  | | **Total** | **7,126** | **1,742** | **24%** |

**^1^** CFR = case fatality rate

Supplementary Table S3 Incidence rates of culture-confirmed melioidosis in Thailand from 2012 to 2015

| **Region** | **Total number of population-year at risk** | **Number**  **of**  **hospitals (provinces)^2^** | **Number of cases ^1^** | | | | | **Incidence rates (per 100,000 population per year) ^1^** | | | | |
| --- | --- | --- | --- | --- | --- | --- | --- | --- | --- | --- | --- | --- |
|  |  |  | **2012** | **2013** | **2014** | **2015** | **Total** | **2012** | **2013** | **2014** | **2015** | **Total** |
| **Central** | 45,153,737 | 21 (17) | 112 | 142 | 155 | 127 | **536** | 0.99 | 1.33 | 1.32 | 1.10 | **1.19** |
| **East** | 13,290,307 | 5 (5) | 85 | 84 | 113 | 82 | **364** | 2.49 | 2.59 | 3.43 | 2.46 | **2.74** |
| **North** | 21,759,235 | 9 (8) | 99 | 93 | 85 | 81 | **358** | 1.99 | 1.71 | 1.49 | 1.44 | **1.65** |
| **Northeast** | 62,745,784 | 17 (17) | 1,332 | 1,359 | 1,481 | 1,303 | **5,475** | 8.25 | 8.94 | 9.45 | 8.29 | **8.73** |
| **South** | 29,423,712 | 12 (10) | 97 | 75 | 95 | 107 | **374** | 1.34 | 1.02 | 1.28 | 1.44 | **1.27** |
| **West** | 8,242,113 | 6 (4) | 10 | 4 | 3 | 2 | **19** | 0.37 | 0.22 | 0.16 | 0.11 | **0.23** |
| **Total** | 180,614,888 | **70 (61)** | 1,735 | 1,757 | 1,932 | 1,702 | **7,126** | 3.79 | 4.02 | 4.23 | 3.74 | **3.95** |

**^1^** Eight provinces had the data obtained from more than one hospital; including, Lopburi (2), Phayao (2), Phang Nga (2), Ratchaburi (3), Saraburi (2), Singburi (2), Songkhla (2) and Suphanburi (2).

**^2^** Of 70 provincial or regional hospitals included in the study, 65, 58, 64 and 62 provided data for year 2012, 2013, 2014 and 2015, respectively.

Supplementary Table S4 Baseline characteristics of 7,126 culture-confirmed melioidosis cases in Thailand from 2012 to 2015

| **Baseline characteristics** | **All cases** | | **Northeast** | **Central** | | **East** | | **North** | | **South** | | **West** | **P value** |
| --- | --- | --- | --- | --- | --- | --- | --- | --- | --- | --- | --- | --- | --- |
|  | **(N=7126)** | | **(n=5475)** | **(n=536)** | | **(n=364)** | | **(n=358)** | | **(n=374)** | | **(n=19)** |  |
| **Gender** |  | |  |  | |  | |  | |  | |  |  |
| • Female | 2287 (32.1%) | | 1788 (32.7%) | 152 (28.4%) | | 125 (34.3%) | | 108 (30.2%) | | 107 (28.6%) | | 7 (36.8%) | 0.16 |
| • Male | 4839 (67.9%) | | 3687 (67.3%) | 384 (71.6%) | | 239 (65.7%) | | 250 (69.8%) | | 267 (71.4%) | | 12 (63.2%) |  |
| **Age group (years)** |  | |  |  | |  | |  | |  | |  |  |
| • <1 to 14 | 264 (3.7%) | | 219 (4.0%) | 13 (2.4%) | | 12 (3.3%) | | 4 (1.1%) | | 16 (4.3%) | | 0 (0.0%) | <0.001 |
| • 15 to 29 | 253 (3.6%) | | 176 (3.2%) | 20 (3.7%) | | 18 (4.9%) | | 9 (2.5%) | | 28 (7.5%) | | 2 (10.5%) |  |
| • 30 to 45 | 1263 (17.7%) | | 972 (17.8%) | 91 (17.0%) | | 74 (20.3%) | | 31 (8.7%) | | 89 (23.8%) | | 6 (31.6%) |  |
| • 45 to 64 | 3874 (54.4%) | | 3049 (55.7%) | 261 (48.7%) | | 166 (45.6%) | | 221 (61.7%) | | 169 (45.2%) | | 8 (42.1%) |  |
| • ≥65 | 1466 (20.6%) | | 1053 (19.2%) | 151 (28.2%) | | 94 (25.8%) | | 93 (26.0%) | | 72 (19.3%) | | 3 (15.8%) |  |
| **Comorbidities^1^** |  | |  |  | |  | |  | |  | |  |  |
| • Diabetes mellitus | 3045 (42.7%) | | 2428 (44.3%) | 190 (35.4%) | | 164 (45.1%) | | 74 (20.7%) | | 180 (48.1%) | | 9 (47.4%) | <0.001 |
| • Hypertension | 1063 (14.9%) | | 764 (14.0%) | 88 (16.4%) | | 93 (25.5%) | | 51 (14.2%) | | 64 (17.1%) | | 3 (15.8%) | <0.001 |
| • Chronic kidney disease | 815 (11.4%) | | 688 (12.6%) | 35 (6.5%) | | 38 (10.4%) | | 27 (7.5%) | | 26 (7.0%) | | 1 (5.3%) | <0.001 |
| • Liver disease | 661 (9.3%) | | 513 (9.4%) | 49 (9.1%) | | 55 (15.1%) | | 17 (4.7%) | | 27 (7.2%) | | 0 (0.0%) | <0.001 |
| • COPD | 200 (2.8%) | | 117 (2.1%) | 27 (5.0%) | | 24 (6.6%) | | 17 (4.7%) | | 13 (3.5%) | | 2 (10.5%) | <0.001 |
| • HIV | 67 (0.9%) | | 44 (0.8%) | 7 (1.3%) | | 7 (1.9%) | | 2 (0.6%) | | 6 (1.6%) | | 1 (5.3%) | 0.04 |
| • Malignancy | 196 (2.8%) | | 128 (2.3%) | 28 (5.2%) | | 20 (5.5%) | | 9 (2.5%) | | 9 (2.4%) | | 2 (10.5%) | <0.001 |
| • Pulmonary tuberculosis | 217 (3.0%) | | 165 (3.0%) | 17 (3.2%) | | 12 (3.3%) | | 6 (1.7%) | | 17 (4.5%) | | 0 (0.0%) | 0.32 |
| • Thalassaemia | 151 (2.1%) | | 117 (2.1%) | 10 (1.9%) | | 6 (1.6%) | | 7 (2.0%) | | 11 (2.9%) | | 0 (0.0%) | 0.82 |
| **Clinical manifestations**^1^ |  |  | | |  | |  | |  | |  | |  |
| • Bacteraemia^2^ | 4910 (68.9%) | | 3741 (68.3%) | 348 (64.9%) | | 286 (78.6%) | | 256 (71.5%) | | 267 (71.4%) | | 12 (63.2%) | <0.001 |
| • Pneumonia^3^ | 2705 (38.0%) | | 2082 (38.0%) | 237 (44.2%) | | 166 (45.6%) | | 93 (26.0%) | | 124 (33.2%) | | 3 (15.8%) | <0.001 |
| • Bacteriuria^4^ | 341 (4.8%) | | 252 (4.6%) | 31 (5.8%) | | 14 (3.8%) | | 26 (7.3%) | | 15 (4.0%) | | 3 (15.8%) | 0.03 |
| • Hepatosplenic abscess^1^ | 580 (8.1%) | | 491 (9.0%) | 31 (5.8%) | | 20 (5.5%) | | 13 (3.6%) | | 24 (6.4%) | | 1 (5.3%) | <0.001 |
| • Septic arthritis^1^ | 385 (5.4%) | | 318 (5.8%) | 27 (5.0%) | | 13 (3.6%) | | 12 (3.4%) | | 14 (3.7%) | | 1 (5.3%) | 0.10 |
| • Osteomyelitis^1^ | 63 (0.9%) | | 40 (0.7%) | 6 (1.1%) | | 3 (0.8%) | | 3 (0.8%) | | 9 (2.4%) | | 2 (10.5%) | <0.001 |

^1^ Comorbidities and clinical manifestations identified by using ICD-10 codes listed in Supplementary Table 1.

^2^ Blood culture positive for *B. pseudomallei*.

^3^ Using ICD-10 codes or sputum culture positive for *B. pseudomallei*.

^4^ Urine culture positive for *B. pseudomallei*

Supplementary Table S5 Factors associated with 30-day mortality in 7,126 culture-confirmed melioidosis cases in 2012-2015 using the univariable logistic model stratified by hospital

| **Baseline characteristics** | **All cases** | **Died** | **Survived** | **Crude odds ratio (95% CI)** | **P value** |
| --- | --- | --- | --- | --- | --- |
|  | (N=7126) | (n=2805) | (n=4321) |  |  |
| **Gender** |  |  |  |  |  |
| • Female | 2287 (32.1%) | 897 (32.0%) | 1390 (32.2%) | 1 | 0.88 |
| • Male | 4839 (67.9%) | 1908 (68.0%) | 2931 (67.8%) | 1.01 (0.91-1.12) |  |
| **Age group (years)** |  |  |  |  |  |
| • <1to14 | 264 (3.7%) | 28 (1.0%) | 236 (5.5%) | 1 | <0.001 |
| • 15-29 | 253 (3.6%) | 78 (2.8%) | 175 (4.1%) | 3.77 (2.34-6.07) |  |
| • 30-45 | 1263 (17.7%) | 459 (16.4%) | 804 (18.6%) | 4.84 (3.22-7.30) |  |
| • 45-64 | 3874 (54.4%) | 1528 (54.5%) | 2346 (54.3%) | 5.56 (3.73-8.28) |  |
| • ≥65 | 1466 (20.6%) | 709 (25.3%) | 757 (17.5%) | 8.22 (5.47-12.35) |  |
| **Comorbidities^1^** |  |  |  |  |  |
| • Diabetes mellitus | 3045 (42.7%) | 1061 (37.8%) | 1984 (45.9%) | 0.67 (0.61-0.74) | <0.001 |
| • Hypertension | 1063 (14.9%) | 428 (15.3%) | 635 (14.7%) | 1.04 (0.91-1.19) | 0.56 |
| • Chronic kidney disease | 815 (11.4%) | 410 (14.6%) | 405 (9.4%) | 1.67 (1.44-1.94) | <0.001 |
| • COPD | 200 (2.8%) | 90 (3.2%) | 110 (2.5%) | 1.31 (0.99-1.75) | 0.06 |
| • Pulmonary tuberculosis | 217 (3.0%) | 97 (3.5%) | 120 (2.8%) | 1.26 (0.96-1.66) | 0.10 |
| • HIV | 67 (0.9%) | 31 (1.1%) | 36 (0.8%) | 1.32 (0.81-2.15) | 0.26 |
| • Liver disease | 661 (9.3%) | 371 (13.2%) | 290 (6.7%) | 2.12 (1.80-2.50) | <0.001 |
| • Malignancy | 196 (2.8%) | 95 (3.4%) | 101 (2.3%) | 1.48 (1.11-1.97) | 0.008 |
| • Thalassaemia | 151 (2.1%) | 36 (1.3%) | 115 (2.7%) | 0.49 (0.33-0.72) | <0.001 |
| **Clinical manifestations^1^** | |  |  |  |  |
| • Bacteraemia^2^ | 2705 (38.0%) | 1574 (56.1%) | 1131 (26.2%) | 3.75 (3.38-4.16) | <0.001 |
| • Pneumonia^3^ | 4910 (68.9%) | 2391 (85.2%) | 2519 (58.3%) | 4.18 (3.70-4.73) | <0.001 |
| • Bacteriuria^4^ | 341 (4.8%) | 209 (7.5%) | 132 (3.1%) | 2.70 (2.15-3.38) | <0.001 |
| • Hepatosplenic abscess^5^ | 580 (8.1%) | 100 (3.6%) | 480 (11.1%) | 0.29 (0.23-0.36) | <0.001 |
| • Septic arthritis^5^ | 385 (5.4%) | 85 (3.0%) | 300 (6.9%) | 0.41 (0.32-0.52) | <0.001 |
| • Osteomyelitis^5^ | 63 (0.9%) | 6 (0.2%) | 57 (1.3%) | 0.16 (0.07-0.38) | <0.001 |
| **Region** |  |  |  |  |  |
| • Northeast | 5475 (76.9%) | 2190 (78.1%) | 3285 (76.0%) | 1 | 0.03 |
| • Central | 536 (7.5%) | 215 (7.7%) | 321 (7.4%) | 1.06 (0.81-1.39) |  |
| • East | 364 (5.1%) | 158 (5.6%) | 206 (4.8%) | 1.21 (0.88-1.68) |  |
| • North | 358 (5.0%) | 111 (4.0%) | 247 (5.7%) | 0.74 (0.53-1.02) |  |
| • South | 374 (5.3%) | 129 (4.6%) | 245 (5.7%) | 0.83 (0.63-1.11) |  |
| • West | 19 (0.3%) | 2 (0.1%) | 17 (0.4%) | 0.18 (0.04-0.85) |  |

**^1^** Comorbidities identified by using ICD-10 codes listed in Supplementary Table 1.

**^2^** Blood culture positive for *B. pseudomallei*.

**^3^** Using ICD-10 codes or sputum culture positive for *B. pseudomallei*.

**^4^** Urine culture positive for *B. pseudomallei.*

**^5^** Using ICD-10 codes*.*

Supplementary Table S6 Factors associated with in-hospital mortality using the univariable logistic model stratified by hospital

| **Baseline characteristics** | **All cases (N=7126)** | **In-hospital**  **mortality (n=1742)** | **Survival discharge (n=5384)** | **Crude odds ratio (95%CI)** | **P value** |
| --- | --- | --- | --- | --- | --- |
| **Gender** |  |  |  |  |  |
| • Female | 2287 (32.1%) | 577 (33.1%) | 1710 (31.8%) | 1 | 0.27 |
| • Male | 4839 (67.9%) | 1165 (66.9%) | 3674 (68.2%) | 0.94 (0.83-1.05) |  |
| **Age group (years)** |  |  |  |  |  |
| • <1 to 14 | 264 (3.7%) | 24 (1.4%) | 240 (4.5%) | 1 | <0.001 |
| • 15 to 29 | 253 (3.6%) | 54 (3.1%) | 199 (3.7%) | 2.70 (1.60-4.55) |  |
| • 30 to 45 | 1263 (17.7%) | 309 (17.7%) | 954 (17.7%) | 3.34 (2.14-5.20) |  |
| • 45 to 64 | 3874 (54.4%) | 946 (54.3%) | 2928 (54.4%) | 3.48 (2.26-5.36) |  |
| • >=65 | 1466 (20.6%) | 409 (23.5%) | 1057 (19.6%) | 4.03 (2.59-6.26) |  |
| **Comorbidities^1^** |  |  |  |  |  |
| • Diabetes mellitus | 3045 (42.7%) | 654 (37.5%) | 2391 (44.4%) | 0.74 (0.66-0.84) | <0.001 |
| • Hypertension | 1063 (14.9%) | 256 (14.7%) | 807 (15.0%) | 0.92 (0.78-1.07) | 0.28 |
| • Chronic kidney disease | 815 (11.4%) | 212 (12.2%) | 603 (11.2%) | 1.13 (0.95-1.34) | 0.17 |
| • COPD | 200 (2.8%) | 64 (3.7%) | 136 (2.5%) | 1.35 (0.99-1.84) | 0.06 |
| • Pulmonary tuberculosis | 217 (3.0%) | 62 (3.6%) | 155 (2.9%) | 1.22 (0.90-1.66) | 0.20 |
| • HIV | 67 (0.9%) | 19 (1.1%) | 48 (0.9%) | 1.15 (0.67-1.99) | 0.61 |
| • Liver disease | 661 (9.3%) | 231 (13.3%) | 430 (8.0%) | 1.75 (1.47-2.08) | <0.001 |
| • Malignancy | 196 (2.8%) | 42 (2.4%) | 154 (2.9%) | 0.80 (0.56-1.14) | 0.22 |
| • Thalassaemia | 151 (2.1%) | 20 (1.1%) | 131 (2.4%) | 0.46 (0.29-0.75) | 0.002 |
| **Clinical manifestations** |  |  |  | |  |
| • Bacteraemia^2^ | 4910 (68.9%) | 1478 (84.8%) | 3432 (63.7%) | 3.40 (2.93-3.94) | <0.001 |
| • Pneumonia^3^ | 2705 (38.0%) | 1046 (60.0%) | 1659 (30.8%) | 3.60 (3.20-4.04) | <0.001 |
| • Bacteriuria^4^ | 341 (4.8%) | 137 (7.9%) | 204 (3.8%) | 2.18 (1.73-2.74) | <0.001 |
| • Hepatosplenic abscess^5^ | 580 (8.1%) | 52 (3.0%) | 528 (9.8%) | 0.29 (0.22-0.39) | <0.001 |
| • Septic arthritis^5^ | 385 (5.4%) | 52 (3.0%) | 333 (6.2%) | 0.48 (0.35-0.65) | <0.001 |
| • Osteomyelitis^5^ | 63 (0.9%) | 4 (0.2%) | 59 (1.1%) | 0.21 (0.07-0.58) | 0.003 |
| **Regions** |  |  |  |  |  |
| • Northeast | 5475 (76.8%) | 1196 (68.7%) | 4279 (79.5%) | 1 | <0.001 |
| • Central | 536 (7.5%) | 196 (11.3%) | 340 (6.3%) | 2.15 (1.55-2.99) |  |
| • East | 364 (5.1%) | 161 (9.2%) | 203 (3.8%) | 2.75 (1.84-4.12) |  |
| • North | 358 (5.0%) | 76 (4.4%) | 282 (5.2%) | 0.97 (0.64-1.47) |  |
| • South | 374 (5.2%) | 110 (6.3%) | 264 (4.9%) | 1.41 (0.99-1.99) |  |
| • West | 19 (0.3%) | 3 (0.2%) | 16 (0.3%) | 0.65 (0.16-2.56) |  |

**^1^** Comorbidities identified by using ICD-10 codes listed in Supplementary Table 1.

**^2^** Blood culture positive for *B. pseudomallei*.

**^3^** Using ICD-10 codes or sputum culture positive for *B. pseudomallei*.

**^4^** Urine culture positive for *B. pseudomallei.*

**^5^** Using ICD-10 codes*.*

Supplementary Table S7 Factors associated with in-hospital mortality by the final multivariable logistic regression model stratified by hospital

| **Baseline characteristics** | **Adjusted odds ratio (95%CI)** | | | **P value** |
| --- | --- | --- | --- | --- |
| **Gender (male)** | 0.82 (0.72-0.93) | | | 0.003 |
| **Age (years)** | 1.00 (1.00-1.01) | | | 0.16 |
| **Comorbidities^1^** |  | | |  |
| • Diabetes mellitus | 0.69 (0.60-0.78) | | | <0.001 |
| • Liver disease | 1.52 (1.26-1.84) | | | <0.001 |
| • Thalassaemia | 0.53 (0.32-0.90) | | | 0.02 |
| **Clinical manifestations** | |  |  |  |
| • Bacteraemia^2^ | 4.07 (3.48-4.77) | | | <0.001 |
| • Pneumonia^3^ | 3.72 (3.28-4.21) | | | <0.001 |
| • Bacteriuria^4^ | 2.36 (1.83-3.05) | | | <0.001 |
| • Hepatosplenic abscess^5^ | 0.36 (0.27-0.49) | | | <0.001 |
| • Septic arthritis^5^ | 0.72 (0.52-0.99) | | | 0.04 |
| • Osteomyelitis^5^ | 0.39 (0.13-1.14) | | | 0.09 |
| **Regions** |  | | |  |
| • Northeast | 1 | | | <0.001 |
| • Central | 2.26 (1.56-3.27) | | |  |
| • East | 2.51 (1.59-3.94) | | |  |
| • North | 0.93 (0.59-1.48) | | |  |
| • South | 1.55 (1.05-2.29) | | |  |
| • West | 0.97 (0.23-4.14) | | |  |

**^1^** Comorbidities identified by using ICD-10 codes listed in Supplementary Table 1.

**^2^** Blood culture positive for *B. pseudomallei*.

**^3^** Using ICD-10 codes or sputum culture positive for *B. pseudomallei*.

**^4^** Urine culture positive for *B. pseudomallei.*

**^5^** Using ICD-10 codes*.*
